# Somatic Genomic Variations in Extra-Embryonic Tissues

**DOI:** 10.2174/138920210793175994

**Published:** 2010-09

**Authors:** Jingly F. Weier, Christy Ferlatte, Heinz-Ulli G. Weier

**Affiliations:** 1 University of California (UC), San Francisco, CA, USA; 2 Department of Cancer & DNA Damage Responses, Life Sciences Division, UC-LBNL, Berkeley, CA, USA

**Keywords:** Gestation, placenta, uterine invasion, cytotrophoblast, aneuploidy, fluorescence in situ hybridization.

## Abstract

In the mature chorion, one of the membranes that exist during pregnancy between the developing fetus and mother, human placental cells form highly specialized tissues composed of mesenchyme and floating or anchoring villi. Using fluorescence in situ hybridization, we found that human invasive cytotrophoblasts isolated from anchoring villi or the uterine wall had gained individual chromosomes; however, chromosome losses were detected infrequently. With chromosomes gained in what appeared to be a chromosome-specific manner, more than half of the invasive cytotrophoblasts in normal pregnancies were found to be hyperdiploid. Interestingly, the rates of hyperdiploid cells depended not only on gestational age, but were strongly associated with the extraembryonic compartment at the fetal-maternal interface from which they were isolated. Since hyperdiploid cells showed drastically reduced DNA replication as measured by bromodeoxyuridine incorporation, we conclude that aneuploidy is a part of the normal process of placentation potentially limiting the proliferative capabilities of invasive cytotrophoblasts. Thus, under the special circumstances of human reproduction, somatic genomic variations may exert a beneficial, anti-neoplastic effect on the organism.

## INTRODUCTION

With an incidence of one in every 5-6 clinically recognized pregnancies, spontaneous abortions (SABs) during the first trimester are the most frequent and often severe pregnancy complication in women [1]. Causes of SABs have been identified as chromosomal abnormalities, uterine defects, immunological problems, hormonal imbalance and infection [2-6]. While more than half of all first trimester SABs are associated with chromosomal abnormalities, nearly 40% remain unexplained [6]. With no apparent association between placental villous morphology and fetal chromosomal abnormalities, SABs with either euploid or aneuploid conceptuses demonstrated incomplete cytotrophoblast (CTB) differentiation and compromised invasion [7-9]. These observations prompted our studies of the chromosomal make-up of extra-embryonic cells at materno-embryonic and fetal-maternal interfaces, i.e., the human placenta and the uterine wall. 

**Fig. (1):**
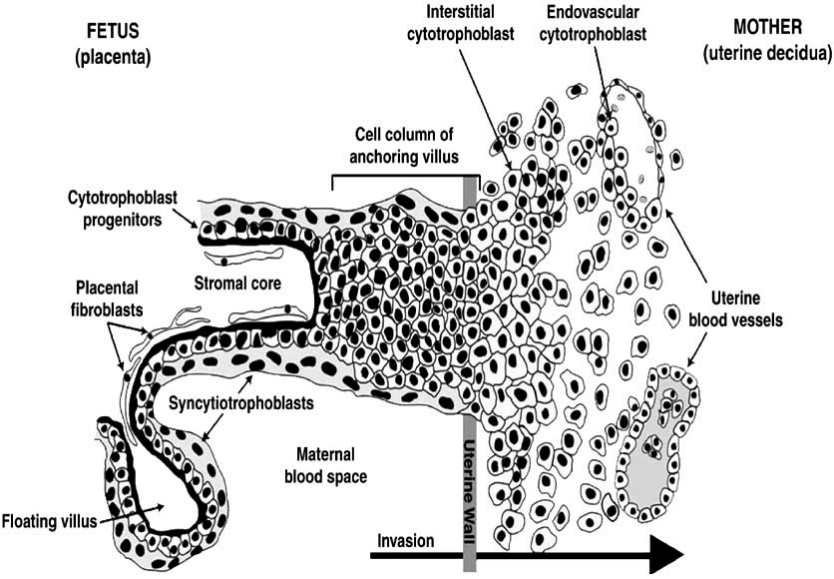
**The different cell types at the fetal-maternal interface around 10-12 weeks of gestation.** Depicted is a diagram of a longitudinal section of an anchoring chorionic villus at the fetal-maternal interface midway through human pregnancy. The anchoring villus functions as a bridge between the fetal and maternal compartments, whereas floating villi are suspended in the intervillous space and are immersed in maternal blood. Cytotrophoblasts in anchoring villi form cell columns and invade the uterine interstitium and maternal blood vessels, thereby anchoring the fetus to the mother and accessing the maternal circulation. (Reprinted from Developmental Biology, vol. 279, JF Weier *et al.*, Human cytotrophoblasts acquire aneuploidies as they differentiate to an invasive phenotype, pages 420-432, Copyright 2005, with permission from Elsevier).

Today, all non-placental species of Eutheria are extinct. The development of eutherian embryos thus depends on the placenta, a transient, but vital organ; thus, the name ‘Placentalia’ [10, 11]. The critical role of the placenta in demonstrating an embryo’s ability to generate lineages of differentiative capacity is illustrated strikingly by the placental abnormalities discovered first in genetically engineered mice [12, 13]. During early human development, cells of the blastocyst are allocated to either the inner cell mass, which later becomes the fetus, or the trophoblast lineage, which forms the outer layer of the chorion and gives rise to the placenta. Soon, specialized functional adaptations of the trophoblast cells will emerge. The chorionic villi are at first small and non-vascular, and consist of the trophoblast only. But soon they increase in size and branch out, whereas the mesoderm carrying branches of the umbilical vessels grows into them and, in this way, vascularizes the villi [14]. To build floating villi, cytotrophoblast cells (CTBs) fuse to form multinucleated syncytial coverings. These villi are attached to the placenta at only one end. The remainder of the floating villus remains immersed in maternal blood to optimize the exchange of gases, nutrients and waste between the mother and fetus (Fig. **1**). In the process of building anchoring villi, CTBs detach from the basement membrane and form columns of mononuclear cells that grow rapidly and invade the uterus. Attached to the fetal portion of the placenta at one end and to the uterine basal plate at the other end, these villi provide anchors of the embryo to the uterine wall. Also of major functional importance, invasive CTBs (iCTBs) rapidly traverse most of the uterine parenchyma. Then, extravillous trophoblasts breach the uterine veins and arteries, and remodel the spiral arteries by replacing the endothelium with cytotrophoblasts, and thereby diverting uterine blood flow to the floating villi [15-19].

In human reproduction, CTBs seem to fulfill several important functions and one would not expect much tolerance of chromosome abnormalities. The cells’ expression of functionally relevant molecules is precisely modulated as they invade the uterine wall or the extracellular matrix [20, 21], and some of the key molecular aspects of CTB differentiation and invasion are known [12, 13]. Except for the fact that invasion is limited to the inner third of the myometrium [19] (Fig. **1**), this process resembles more tumorigenesis than organ development. Remarkably, months of rapid placental growth and CTB invasion will bring large numbers of CTB cells into the myometrium [17, 19]. Yet, in most pregnancies, the uterus contracts after labor and delivery, and progressively resumes normal size, shape and function without any signs of unscheduled cellular growth, presence of fetal cells or neoplasm.

### Ontogenetic Aspects and Cytogenetics Underlying Cytotrophoblast Function in the Normal Pregnancy 

In most pregnancies, the fetus and the placenta have the same chromosomal complement, because both structures are descendants of the same zygote. The known exceptions are mosaics caused by chromosomal instability [22]. However, in one to two percent of viable pregnancies, chorionic villus sampling around 10 to 12 weeks of gestation reveals a numerical chromosome abnormality, most often trisomy, confined to the placenta [22, 23]. Confined placental mosaicism (CPM), as it has been termed, can occur as a result of postzygotic errors in mitosis, in which case the conceptus often retains a normal karyotype. Alternatively, a trisomic blastocyst may be rescued by chromosome loss within the embryo, leaving the extraembryonic lineages and thus the placenta trisomic [24-26]. Approximately 20% of pregnancies complicated by idiopathic intrauterine growth restriction are associated with CPM [27-29]. About 5% of conceptions with trisomy 13 or 18 develop trophoblast-confined mosaicism and continue their intrauterine development into the third trimester [30]. Placentae from non-mosaic newborns with trisomy 13 or 18 or stillborns have shown trophoblast-confined diploid/trisomy mosaicism [31]. Thus, the process of ‘trisomic zygote rescue’ might allow some trisomic conceptuses to acquire a partially functional placenta and survive into the third trimester, if not to term. These observations emphasize the important issue that fetal survival and maternal health are not always dependent on the fetal karyotype. Proper function of extra-embryonic tissues such as the placenta or chorionic villi, are needed for embryonic/fetal survival, and genetic or morphological abnormalities in these tissues might adversely affect fetal development [19].

In many instances, the level of mosaicism detected by chorionic villus sampling does not properly reflect the level in the term placenta as a whole, which may vary considerably [32]. To date, most genetic studies have examined cells in the floating villi including the trophoblast populations. On the other hand, very little is known about the karyotypes of human CTBs that arise from anchoring villi and subsequently invade the uterine wall. Interestingly, the analogous population of invasive trophoblasts in mice is believed to undergo endoreduplication [33, 34]. A few additional reports partly based on cytometry results suggested the possibility that human iCTBs have an elevated ploidy level (hypertetraploid and hyperoctaploid) [35-37]. 

In our studies of uncomplicated pregnancies, we did find that a majority of iCTBs identified as human leukocyte antigen – G (HLA-G) positive cells [21] were chromosomally abnormal [38-40]. Please note that this population is rarely studied by any means, because of the difficulty of obtaining these samples. Thus, it is not surprising that this phenomenon was not described earlier. Our work showed that in the CTB differentiation pathway, fate specification and cell cycle entry are tightly coordinated. Specifically, CTBs undergo a final mitotic cycle as they enter the cell columns (Fig. **1**), i.e., the structures that bridge the gap between the chorionic villi and decidua. Subsequently, uterine invasion is coordinated with permanent withdrawal from the cell cycle [41, 42].

### Multicolor FISH and Karyotype Analysis of Interphase Cells 

**Fig. (2):**
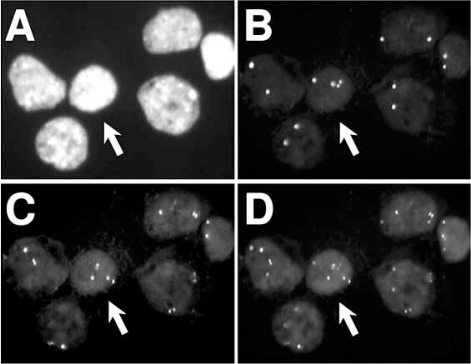
**FISH analysis of isolated CTBs reveal numerical chromosome aberrations.** The six cell nuclei shown in this panel were hybridized with a triple probe combination and counterstained with DAPI. The probes used were CEP 16 (Spectrum Green), CEP X (Spectrum Orange) and CEP Y (Spectrum Aqua, blue) (Abbott, Inc.). Since these CTBs were isolated from extraembryonic tissue of a female conceptus, the nuclei did not show any blue signals. The panels show the DAPI, CEP X and CEP 16 signals in panels (**A**), (**B**), and (**C**), respectively. The composite image is shown in panel (**D**). The arrows point at an aneuploid nucleus showing 4 red and 4 green signals. Please see Weier *et al.* [39] for further examples.

We have found that a subset of freshly isolated CTBs from normal pregnancies have numerical chromosomal abnormalities [39]. In our experiments, we applied a variety of non-isotopically labeled DNA either prepared in-house [43-47] or obtained commercially (Abbott Molecular, Downers Grove, IL). With few exceptions, probes used in our studies were either DNA repeat probes or locus-specific DNA probes [48-51]. When cells obtained from patients with uncomplicated pregnancies were hybridized with three chromosome enumerator probes (CEPs) specific for the X- and Y-chromosomes and chromosome 16 (CEP X, CEP Y, and CEP 16, respectively) or chromosomes 13, 18 and 21, we observed that many CTBs displayed a continuum of CEP X signals that ranged from closely spaced pairs to widely separated CEP X signals (Fig. **2**) [39,40]. 

**Fig. (3):**
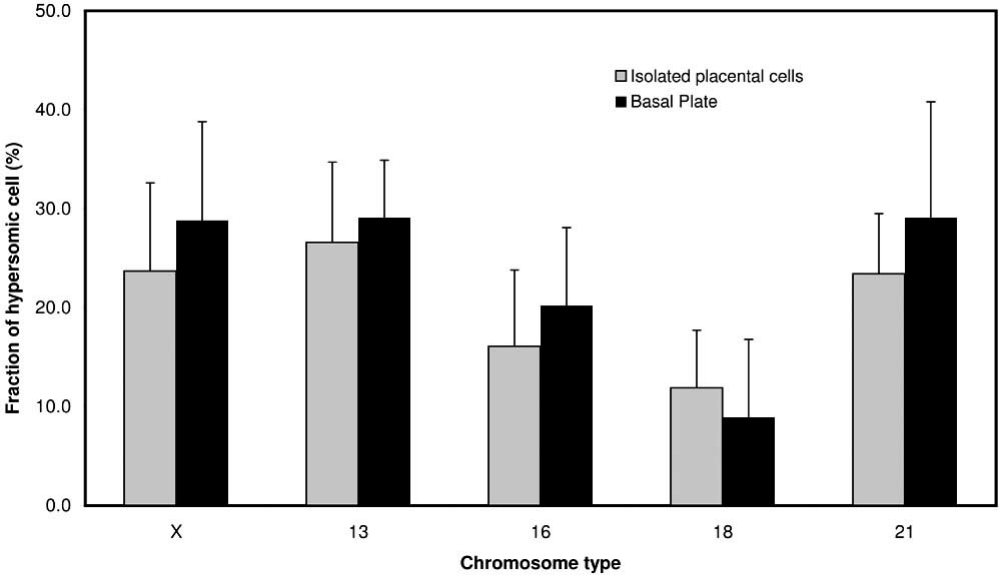
**FISH analysis of isolated CTBs obtained from women with normal pregnancies.** For most of the chromosomes, the average fraction hypersomic cells of isolated from the placenta is slightly lower than that of cells from the basal plate (i.e., uterine wall). Six chromosomes (13,16,18,21,X-chromosome,Y-chromosome) were scored. Due to the mix of female and male samples, the Y-chromosome data was excluded from analysis.

**Fig. (4):**
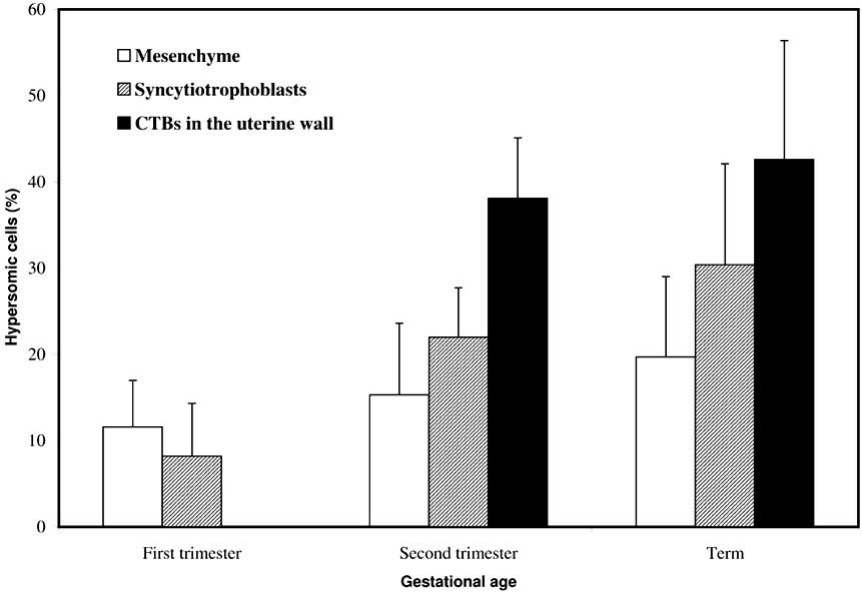
**FISH analysis of invasive CTBs in at the fetal-maternal interface.** Analysis of tissue sections allowed calculation of the percentage of hyperdiploid cells in the various placental compartments during the first and second trimesters and at term. Samples of CTBs embedded in the uterine wall could not be obtained during the first trimester. During the second trimester, hyperdiploid cells were found more often within the uterine wall than in association with the floating villi (* p < 0.002).

In most cells, the copy number of X-chromosome was greater than the copy number of chromosome 16 or 18, but we did not observe significant differences between the fraction of hypersomic cells isolated from either unselected placentae or the basal plate (Fig. **3**). Since the subpopulation of replicating, presumably diploid, CTB progenitors is rapidly depleted after the first trimester of pregnancy, we also analyzed aneuploidy as a function of gestational age. The CTBs isolated from first-trimester and term placentas had the lowest and highest mean rates of aneuploidy, respectively (22.2 ± 8.5% vs. 40.5 ± 9.0%), whereas second-trimester cells had an intermediate value (35.8 ± 12.5%) [39]. Furthermore, we studied the chromosomal make-up of CTBs in situ. Frozen tissue sections from three gestational ages were studies, and three cell types were scored: mesenchymal cells in the central cores of the chorionic villi, multinucleated syncytiotrophoblasts that cover these villi, and CTBs within the uterine wall (Fig. **4**). The average rate of hyperdiploidy among mesenchymal cells was 11.6 ± 5.4%, 15.3 ± 8.3%, and 19.3 ± 9.3% in tissue sections of first-trimester, second-trimester and term placentas, respectively. In comparison, syncytiotrophoblasts showed a higher rate that increased with advancing gestational age (8.2 ± 6.1%, first trimester; 22.0 ± 5.7%, second trimester; and 30.4 ± 11.7% at term). Of all the cells that were scored, CTBs in the uterine wall were more likely to be hyperdiploid: 38.1 ± 7.0% of cells in the second trimester and 42.6 ± 13.8% of cells at term had extra chromosomes. Finally, analysis of tissue sections (not shown) showed that the spatial distribution of the aneuploid trophoblasts appeared to be random, suggesting that the cells acquire aneusomies sporadically as opposed to clonal expansion of an aneuploid CTB subset.

We also have shown that the aneuploid cells, which fail to incorporate bromodeoxyuridine (BrdU), are HLA-G positive [39]. Additionally, the fraction of hyperdiploid cells increased with gestational age (Fig. **4**), most likely reflecting the fact that the population of progenitor cells is largely depleted by mid-second trimester as a result of their differentiation to syncytiotrophoblasts or iCTBs. Together these findings suggest that the aberrations in chromosome number in iCTBs arise during the last mitotic cycle, a conclusion that is bolstered by our *in situ* analyses. The sequestration of the aneuploid cells within the uterine wall provides a likely explanation why hyperdiploid CTBs are not found by chorionic villus sampling. 

Most SABs are sporadic and while chromosomal errors are their most prominent cause, the exact mechanism of the abortion event is still in dispute. Qumsiyeh *et al*. [52] suggested a mechanism for aneuploid SAB: increased apoptosis and decreased cell proliferation in chromosomally abnormal placental cells. These investigators found a higher fraction of apoptotic cells among the stromal cells from chromosomally abnormal villi compared to those from chromosomally normal villi. But apoptotic fractions were no different among trophoblastic cells from either type of villus. Moreover, in blood vessel walls, chromosomally abnormal villi had a lower number of proliferative cells than chromosomally normal villi. The authors suggested that apoptosis of stromal cells and cell proliferation in both blood vessels and stromal cell compartments play an important role in the differentiation and function of villi. An abnormal chromosome complement may lead to decreased cell proliferation of both vascular smooth muscle and stromal cells, however there is still no explanation for euploid SABs.

## CONCLUDING REMARKS

The human embryo/fetus cannot develop autonomously. The various portions of the placenta connect the conceptus in many ways to the mother, all of which are critical to the outcome of the pregnancy. Proper formation of the maternofetal interface, i.e., the placenta and villous structures, is essential to ensure normal embryonic growth and fetal development. The results of our studies suggest that chromosomal abnormalities, such as the gain or loss of chromosomes, have functional consequences on the establishment of vasculature and fetal development. We have determined that human CTBs acquire aneuploidies as they differentiate to an invasive phenotype suggesting that aneuploidy is an important component of normal placentation, likely to limit the proliferative and invasive potential of CTBs [39, 40]. It will be very interesting to know, if similar alterations of the genotype of CTBs happen in SABs, which are known to be associated with incomplete CTB differentiation and invasion.

Aneuploid CTBs have been found in large numbers in the placentae and chorionic villi associated with triploid conceptuses as well as in normal, uncomplicated pregnancies [38, 39]. In both instances, the fraction of aneuploid CTBs increases with gestational age, but does not appear to exert a detrimental effect on the organism. This is in contrast to reports of genomic variations in other organs such as the reproductive system, the human brain or in hematological disorders [44, 53-56], where an association between aneuploidy and disease could be established [54, 56-58]. While it seems that all human tissues are affected by somatic genomic variations [59], albeit to a greatly varying extent, aneuploidy might be missed when present in rare cells [57, 60, 61]. This low frequency of genomic variants marks a demand for highly sensitive technologies-of-scale that offer the high throughput necessary to detect such rare events. The application of improved methods for interphase cell analysis [62-64] and the novel genomic and proteomic technology platforms that have been developed in recent years promise to meet this need [65].

## DISCLAIMER

This document was prepared as an account of work sponsored by the United States Government. While this document is believed to contain correct information, neither the United States Government nor any agency thereof, nor The Regents of the University of California, nor any of their employees, makes any warranty, express or implied, or assumes any legal responsibility for the accuracy, completeness, or usefulness of any information, apparatus, product, or process disclosed, or represents that its use would not infringe privately owned rights. Reference herein to any specific commercial product, process, or service by its trade name, trademark, manufacturer, or otherwise, does not necessarily constitute or imply its endorsement, recommendation, or favoring by the United States Government or any agency thereof, or The Regents of the University of California. The views and opinions of authors expressed herein do not necessarily state or reflect those of the United States Government or any agency thereof, or The Regents of the University of California.

## ABBREVIATIONS

BrdU: = Bromodeoxyuridine
CEP: = Chromosome enumerator probe
CPM: = Confined placental mosaicism
CTB: = Cytotrophoblast
iCTB: = Invasive cytotrophoblast
HLA-G: = Human leukocyte antigen - G
SAB: = Spontaneous abortions
